# Isolation and characterization of a native avirulent strain of *Streptococcus suis* serotype 2: a perspective for vaccine development

**DOI:** 10.1038/srep09835

**Published:** 2015-04-20

**Authors:** Xinyue Yao, Ming Li, Jing Wang, Changjun Wang, Dan Hu, Feng Zheng, Xiuzhen Pan, Yinling Tan, Yan Zhao, Liwen Hu, Jiaqi Tang, Fuquan Hu

**Affiliations:** 1 Department of Microbiology, Third Military Medical University, Chongqing, 400038, China; 2 Department of Epidemiology, Research Institute for Medicine of Nanjing Command, Nanjing 210002, China; 3 PLA Research Institute of Clinical Laboratory Medicine, Nanjing general hospital of Nanjing Military Command, Nanjing 210002, China

## Abstract

*Streptococcus suis*, an emerging infectious pathogen, is the cause of two large-scale outbreaks of human streptococcal toxic shock syndrome in China, and has attracted much attention from the scientific community. The genetic basis of its pathogenesis remains enigmatic, and no effective prevention measures have been established. To better understand the virulence differentiation of *S. suis* and develop a promising vaccine, we isolated and sequenced a native avirulent *S. suis* strain (05HAS68). Animal experiments revealed that 05HAS68 is an avirulent strain and could protect piglets from the attack of virulent strains. Comparative genomics analyses demonstrated the genetic basis for the lack of virulence in 05HAS68, which is characterized by the absence of some important virulence-associated factors and the intact 89K pathogenicity island. Lack of virulence was also illustrated by reduced survival of 05HAS68 compared to a virulent strain in pig whole blood. Further investigations revealed a large-scale genomic rearrangement in 05HAS68, which was proposed to be mediated by transposase genes and/or prophages. This genomic rearrangement may have caused the genomic diversity of *S. suis*, and resulted in biological discrepancies between 05HAS68 and highly virulent *S. suis* strains.

S*treptococcus suis* (*S. suis*) is an emerging zoonotic pathogen that causes various life-threatening infections, including meningitis, arthritis, septicemia, and pneumonia, in humans and animals, and is responsible for substantial economic losses in the swine industry^1^,^2^,^3^,^4^. The first human case of *S. suis* infection was diagnosed in Denmark in 1968. Subsequently, *S. suis* has caused sporadic cases of meningitis and sepsis in humans. Based on the antigenicity of capsular polysaccharides (CPS), *S. suis* can be classified into 33 serotypes, of which *S. suis* serotype 2 (SS2) is the most prevalent from human cases worldwide^4^. Two large-scale outbreaks of human infections in China (in Jiangsu and Sichuan Provinces in 1998 and 2005, respectively) induced by SS2 have raised considerable public health concerns^5^,^6^,^7^. Notably, both outbreaks showed a prevalent feature of streptococcal toxic shock syndrome (STSS), which is manifested with acute high fever, multiple organ failure, severe hemorrhage, and high mortality rate^1^,^6^. Two representative highly virulent strains (05ZYH33 and 98HAH12), isolated from patients with STSS, have been investigated for several years by our joint research groups^6^,^7^,^8^,^9^,^10^,^11^,^12^.

Multiple virulence-associated genetic elements have been identified in previous studies. An 89K pathogenicity island (89K PAI) was first identified in the Chinese highly virulent SS2 strains (05ZYH33 and 98HAH12). The 89K PAI can be spontaneously excised to form an extra-chromosomal circular product, and is related to the high pathogenicity of *S. suis*^10^. Previous comparative genomics analyses and conjugative experiments jointly reveal that the 89K PAI can be transferred to recipient strains, making them more virulent^13^. Moreover, analysis using NimbleGen tiling arrays indicates that some virulence-associated factors (VAFs) are closely related to the pathogenesis of *S. suis* infection^12^. The most critical VAF validated in *S. suis* is the CPS, which is considered as an important antiphagocytic factor^14^,^15^. Other VAFs, such as fibronectin binding protein (FBP), muramidase-related protein (MRP), extracellular protein factor (EPF), and suilysin, have been reported to participate in the process of colonization in organs and interactions with host cells or extracellular matrix^16^,^17^,^18^.

However, little is known about the virulence differentiation among diverse *S. suis* strains. Previous studies revealed that the genomic arrangement of *S. suis* is important for its virulence differentiation, and comparative genomics analysis is a helpful way to unravel the underlying genomic reconstruction events. To characterize the genetic basis of virulence variation of *S. suis*, we isolated and sequenced a native avirulent strain of *S. suis* (05HAS68) from the Chinese epidemic region, and explored its biological properties *in vitro* and *in vivo*. Then, we performed comparative genomics analysis and found distinct characteristics of the 05HAS68 genome from the virulent strains, which may explain their differences in biological properties. Moreover, phylogenetic analysis suggested evolutionary relationships of various *S. suis* strains and the genetic basis associated with their virulence.

## Results

### Characteristics of a SS2 strain

The *S. suis* strain in this study (designated 05HAS68) was isolated from the tonsil of a healthy pig in the epidemic area of Jiangsu Province, China, in 2005. Gram staining showed that 05HAS68 is a Gram-positive coccus appearing in short chains under an optical microscope. The capsular substance of 05HAS68 could be clearly observed through a transmission electron microscopy. The biochemical reactions of this strain are in accordance with the *Streptococcus* genus (data not shown). Moreover, 16S rRNA classification and serologic tests indicated that the isolate belongs to the SS2. Multi Locus Sequence Typing (MLST) analysis classified 05HAS68 as sequence type 28 (ST28) (Table 1).

When cultured on sheep blood agar, 05HAS68 formed slightly gray or semitransparent, wet, smooth, and glossy colonies. The strain showed α-hemolysis, as reflected by the ~ 2 mm diameter hemolytic rings on the plates. This finding was further confirmed by measuring hemolytic activities in a photometric assay. The hemolytic titer of 05HAS68 was significantly lower than that of 05ZYH33 (Fig. S1), suggesting that 05HAS68 performs incomplete hemolytic activity in pig blood.

### 05HAS68 is an avirulent *S. suis* strain and exhibits protective effect in a piglet infection model

**Figure 1:**
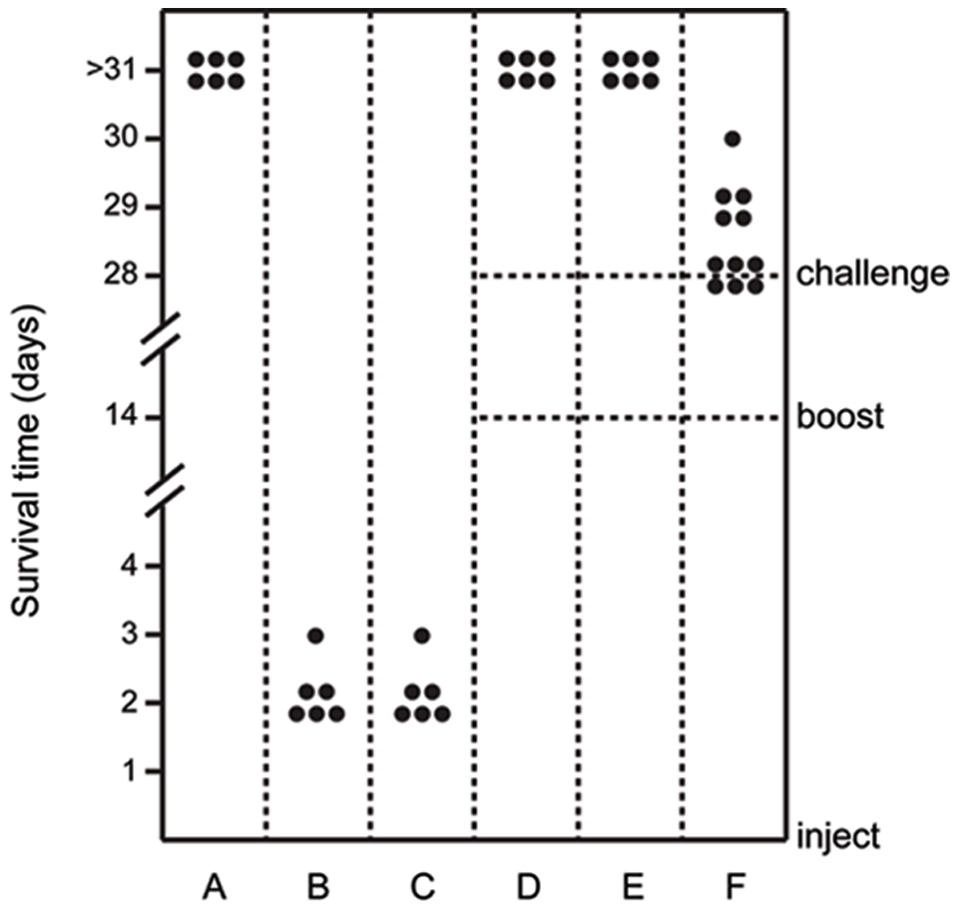
SPF-piglet infection experiments. Each piglet was intravenously injected with a different bacterial suspension avirulent strain (05HAS68), highly virulent strain (05ZYH33 or 98HAH12), or attenuated *S. suis* strain (Δ*salKR*)) at a dose of 10^8^ CFU/piglet. Piglets were given the same dose as a booster at day 14, and then challenged with the highly virulent strains (05ZYH33 or 98HAH12) at day 28 where indicated. Each datum point represents one piglet. Survival time (d) of individual piglet was monitored. (A). Piglets were injected with 05HAS68. (B). Piglets were injected with 05ZYH33. (C). Piglets were injected with 98HAH12. (D and E) Piglets were injected with 05HAS68, and given the same dose for booster at day 14, then challenged with 05ZYH33 (in **Panel D**) or 98HAH12 (in **Panel E**). (F). Piglets were injected with Δ*salKR* and given the same dose for booster at day 14, then challenged with 05ZYH33.

To evaluate the virulence of 05HAS68 *in vivo*, we injected three-week-old specific pathogen-free (SPF) piglets with 05HAS68 or virulent strains. As shown in Figure 1B and 1C, all of the piglets injected with 05ZYH33 or 98HAH12 developed most of the typical disease symptoms, including high fever, poor appetite, limping, shivering, and dyspnea. Five of the piglets died on day 2 after inoculated with 05ZYH33 or 98HAH12, and the last one died on day 3 (Fig. 1B and 1C). In contrast, all six piglets injected with 05HAS68 survived more than one month without any obvious clinical symptoms, such as high fever, dyspnea, limping, or canthus fester (Fig. 1A). These results suggested that 05HAS68 is an avirulent *S. suis* strain.

To preliminarily assess the protective effect induced by 05HAS68 in piglets, we inoculated two groups of piglets with 05HAS68, and administered the same dose as a booster at day 14. Subsequently, each group was challenged with strain 05ZYH33 or 98HAH12 at day 28. All six piglets that were first inoculated with 05HAS68 survived the challenge with 05ZYH33 or 98HAH12 (Fig. 1D and 1E). As a further control, an attenuated SS2 strain (Δ*salKR*) was used to demonstrate the irreplaceability of 05HAS68 in terms of its protective effect. As shown in Figure 1F, all of the Δ*salKR*-injected piglets died in three days after challenged with 05ZYH33. The survival ratio and survival time of the piglets that were initially treated with 05HAS68, were higher and longer, respectively, than those of the piglets initially injected with Δ*salKR*.

### General features of the 05HAS68 genome

**Figure 2:**
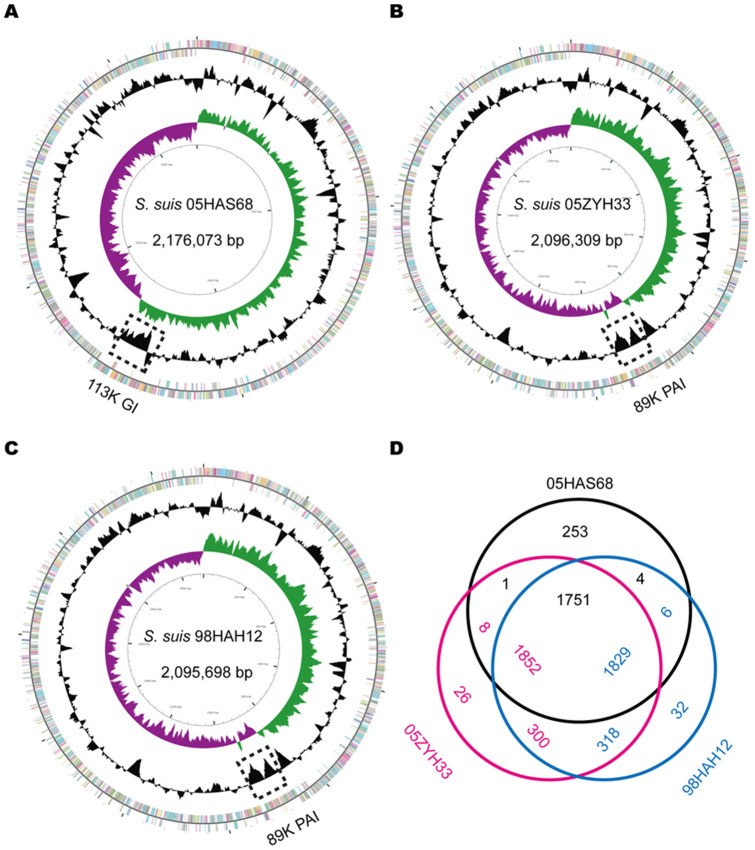
Schematic circular diagrams of the 05HAS68, 05ZYH33, and 98HAH12 genomes. **(**A). Circular map of the 05HAS68 genome completed in the present study. Keys for circular diagrams (from outside to inside): annotated genes are presented as a pair of concentric circles distinguished by different colors in the COG classification (positive strand on the outside, negative strand on the inside); GC content of the 05HAS68 genome; GC skew plot (green for GC skew^+^; purple for GC skew^−^); and scale of the whole genome (bp). Circular maps of the 05ZYH33 (in **Panel B**) and 98HAH12 genomes (in **Panel C**) completed previously and used for comparative genomics analyses in this study. (D). Transformed Venn diagram. The genes of 05HAS68, 05ZYH33, and 98HAH12 were compared by BLASTp. Each strain is represented by one color, and the gene numbers are displayed in the same color. Numbers in the intersectional region indicate genes shared by two or three strains. For example, 1751, 1852, and 1829 represents the gene numbers of 05HAS68, 05ZYH33, and 98HAH12 that are shared by these three strains. Moreover, 253, 26, and 32 is the number of the unique genes in 05HAS68, 05ZYH33, and 98HAH12, respectively. Notably, the numbers in the intersections are slightly different due to the fact that there are multiple duplicate gene copies in the bacterial genome.

To elucidate the genomic properties of 05HAS68, we performed whole-genome sequencing for this strain. Eventually, we successfully assembled a single circular 2,176,073 bp chromosome with 41.16% G + C content (Fig. 2A), 2009 genes, 56 tRNAs, and 12 rRNAs (Table 1). Approximately 87.3% of the genome sequence is coding regions. The annotated sequence is available at the National Center for Biotechnology Information (GenBank accession number: CP006930). As shown in Table 1, the *S. suis* genomes, including 05HAS68 display a relatively similar genome size (ranging from 1.98–2.24 Mbp with a mean size of 2.10 Mbp), average G + C content (ranging from 41.0%–41.4% with an average of 41.2%), and number of protein coding genes (ranging from 1824–2190). However, the gene composition of these genomes varies widely.

We mainly compared 05HAS68 with two highly virulent SS2 strains (05ZYH33 and 98HAH12), which were isolated in the Chinese epidemic region (Fig. 2)^6^,^7^. Bioinformatics analysis indicated that 05HAS68 contains 253 unique genes and shares 1751 common genes with 05ZYH33 and 98HAH12 (Fig. 2D). Among the 253 unique genes, 23 are associated with DNA replication, recombination, and repair, 17 are associated with secretion, and 10 are associated with transcription. Notably, a large number of the unique genes are predicted to be prophage-related genes.

### Large-scale genomic rearrangement in *S. suis* strains

**Figure 3:**
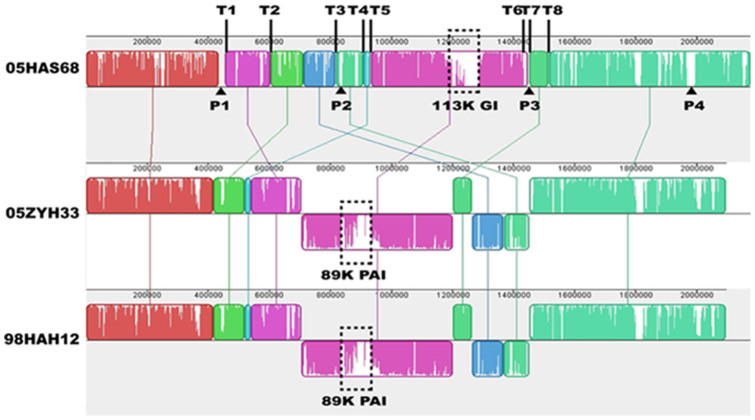
Mauve comparison diagrams of the 05HAS68, 05ZYH33, and 98HAH12 genomes. Each colored region is a locally collinear block (LCB). The LCBs below the genome's center line are in reverse complement orientation compared with the 05HAS68 genome. T1 to T8 indicate the positions of transposase-encoded genes. P1 to P4 represent the positions of prophage-related gene clusters.

Alignment of the whole *S. suis* genome sequences between 05HAS68 and 05ZYH33/98HAH12 showed a large-scale genomic rearrangement in 05HAS68, including deletion, insertion, translocation, and inversion (Fig. 3). This extensive genomic rearrangement occurred across the origins/terminus axis (*ori/ter* axis) of the 05HAS68 genome, which located in the region from 450 kb to 1500 kb. The most evident variations are four translocated segments and a large-scale inverted segment of 05HAS68 (Fig. 3). Interestingly, eight transposase family protein genes are located at the terminals of these rearrangement segments (positioned from T1 to T8).

### Virulence-related elements differ in 05HAS68, 05ZYH33, and 98HAH12

**Figure 4:**
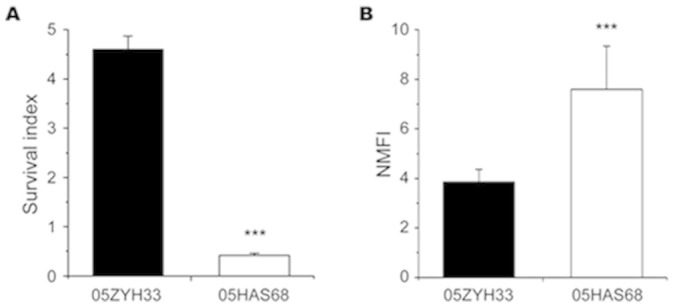
Whole blood killing and bacterial adherence assays. (A). Whole blood killing assay. Data are expressed as survival index: (CFU bacteria at end of assay)/(initial bacteria CFU) with the mean ± SD of three independent experiments. (B). Comparison of bacterial adherence capability between 05HAS68 and 05ZYH33. The normalized mean fluorescence intensities (NMFI) of the cells are shown as columns with the mean ± SD of intracellular bacteria/ml. ***P < 0.001.

In addition to the large-scale genomic rearrangement, we also found some differences in virulence-related elements between 05HAS68 and the highly virulent *S. suis* strains, including some VAFs, two-component signal transduction systems (TCSTSs), and genomic islands (GIs). The 89K PAI was not found in 05HAS68 when compared with 05ZYH33 or 98HAH12 (Fig. 2 and 3). Further, several important VAFs, such as EPF, suilysin, PlcR family transcriptional regulator, and virulence-associated protein E, are absent in 05HAS68 (Table 2). Moreover, virulence-associated TCSTSs, such as SalK/SalR, NisK/NisR, VirR/VirS, and the orphan regulator RevS, are not present in 05HAS68. However, we noticed that several VAF and TCSTS encoding genes are still present in the 05HAS68 genome, such as choline-binding protein D (*cbpD*, *HAS68_1948)*, hemolysin III (*HAS68_1078*), glutamate dehydrogenase (*gdh*, *HAS68_0240*), sortase A (*HAS68_0597*), *ciaRH* (*HAS68_0994*/*HAS68_0995*), *ihK/irr* (*HAS68_1505*/*HAS68_1506*), and orphan regulator *covR* (*HAS68_1571*). We also identified capsule synthesis genes in the genome of 05HAS68, but it seems that the capsule provided weak protective effect to this strain. Using *in vitro* whole blood killing assays, we found that the 05ZYH33 strain proliferated much better than 05HAS68 in pig blood (Fig. 4A), although the cellular adherence assays using Hep-2 cells demonstrated that 05HAS68 has a significantly enhanced adherence capability to the host cell (Fig. 4B).

### Prophage gene clusters and CRISPR structure in 05HAS68

As stated above, the 05HAS68 strain contains 253 unique genes compared with 05ZYH33 and 98HAH12. Of these genes, 69 are predicted to be prophage-related genes, composing four prophage gene clusters (Table S1). Three of the prophage-related gene clusters are located at the terminals of rearrangement segments (positioned from P1 to P3), whereas the other cluster is situated near the end of the chromosome (marked as P4) (Fig. 3). Comparison of these prophage gene clusters using the BLASTp algorithm revealed no similarities with each other. Moreover, there is a clustered regularly interspaced short palindromic repeat (CRISPR) structure in the 05HAS68 genome, consisting of seven 36-bp direct repeats (DRs), seven spacers of different lengths, and five functional proteins (Csn1, integrase, Cas1, Cas2, and Cas7) (Table S1).

### GI in 05HAS68

The 05HAS68 strain has no 89K PAI but a unique 113K GI. This GI is 112,617 bp in length with an average G + C content of 38.37%, which is significantly different from that of the whole genome (41.16%) (Fig. 2). This novel GI is inserted into three 15-bp DRs (GTT ACT CTT AAA TAA), two of these DRs are located at the end of the GI, whereas the remaining DR divides the GI into 63,845 bp and 48,772 bp. The 113K GI is predicted to encode genes that participate in two metabolic pathways, namely, the galactose and L-fucose metabolic pathways.

### Phylogenetic analysis of the 05HAS68 strain

**Figure 5:**
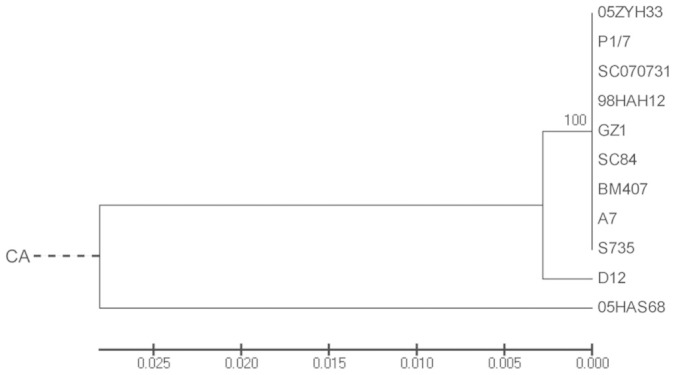
Phylogenetic tree of *S. suis* genomes. Phylogenetic tree showing the relationship of 05HAS68 and other *S. suis* strains based on alignment of the *ITS* gene sequences. CA indicates the assumed common ancestor. The bar indicates the relative evolutionary distance.

To analyze the evolutionary relationship between 05HAS68 and other SS2 strains, we used the internal transcribed spacer (*ITS*) gene sequences of the 16S–23S rRNA gene to construct a phylogenetic tree. As shown in Figure 5, all SS2 strains, except 05HAS68, are located on the top clade of the tree, whereas 05HAS68 is paralleled with *S. suis* D12 (out-group, serotype 9). Moreover, 05HAS68 is more divergent compared with the other SS2 strains.

## Discussion

SS2 had caused two large-scale outbreaks in China, with a relatively high occurrence of STSS and abnormally elevated mortality^6^. The virulence differentiation of *S. suis* strains remains ambiguous, and previous tentative VAF-based vaccines are insufficient to protect hosts against *S. suis*^19^,^20^,^21^. In the current study, we isolated and sequenced an avirulent strain of SS2, named *S. suis* 05HAS68. We assessed some of its biological properties and sequenced its genome, and the results showed great significance to explain the virulence differentiation of *S. suis* strains and to develop a promising vaccine against *S. suis* infection. As reflected by our results, 05HAS68 is a native avirulent SS2 strain, displaying no virulence and exerting a protective effect in the piglet infection experiments. Further genomic comparisons between 05HAS68 and other pathogenic strains revealed valuable information on the possible virulence differentiation among *S. suis* strains.

We found that the VAFs and TCSTSs involved in the main pathogenic processes of *S. suis* differ between virulent and avirulent strains, such as those that participate in colonization, invasion, and dissemination of *S. suis*^16^,^22^. Among these differential genes, suilysin is a quintessential VAF in *S. suis*, contributing to high bacterial density, enhanced inflammation in the brain, and subsequent increase in mortality^18^. 05HAS68 exhibited typical α-hemolysis on pig blood agar, and showed a lower hemolytic activity compared with 05ZYH33 (Fig. S1), which may due to its absence of suilysin. However, 05HAS68 displayed significantly enhanced adherence capability to the Hep-2 cells (Fig. 4B). A plausible explanation of this observation could be that a *Streptococcal* hemagglutinin encoding gene is exclusively detected in 05HAS68 but not in 05ZYH33 or 98HAH12. As we know, hemagglutinin usually contributes to the adherence and colonization of microorganism, and its role in adherence capacity has been identified in diverse bacterial species, such as *Mycobacterium leprae*^23^, *Moraxella catarrhalis*^24^, and *Salmonella enterica*^25^. *Streptococcus* colonization depends upon several biological processes, such as adherence, cell signaling, and host modulation. In GAS, cell surface proteins (such as SpeB) can mediate adherence to glycoproteins and the extracellular matrix components, which may promote the colonization capabilities of bacteria^26^. We speculated that 05HAS68 is more adapted for colonization than 05ZYH33 because of its higher adherence to host cells.

CPS is an important VAF validated in *S. suis*, and the bacteria can be protected by capsule in adverse environment. However, the capsule seems not to protect 05HAS68 from whole blood killing sufficiently (Fig. 4A). We proposed that the capsule structures of 05HAS68 might be more fragile than 05ZYH33, in spite of the existence of capsule synthesis genes. Meanwhile, the hemolytic activity of 05HAS68 was significantly lower than that of 05ZYH33, and some important VAFs are absent in 05HAS68, so we think that these differential characteristics jointly contribute to decreased survival of 05HAS68 in whole blood.

In addition, the absence of several TCSTSs and the entire 89K PAI may be the leading cause of the virulence loss in 05HAS68 (Table 2). An orphan response regulator, RevS, plays an important role in the pathogenesis of *S. suis* infection, and the isogenic knockout mutant of *revS* was shown to be attenuated in the piglet infection experiment^27^. Previous reports demonstrated that the 89K PAI is only present in the Chinese highly virulent SS2 strains, which could confer pathogenicity to its host strain^7^. SalK/SalR and NisK/NisR are located on the 89K PAI of 05ZYH33 and are related to virulence regulation in *S. suis*. SalK/SalR is required for the full virulence of highly pathogenic SS2 in China^8^, whereas NisK/NisR is important in the process of colonization and invasion^28^. Furthermore, comparative genomics analyses revealed the absence of the 89K PAI in 05HAS68, and the presence of a novel 113K GI in its genome (Fig. 2 and 3). Two predicted sugar metabolic pathways, the galactose and L-fucose pathway, are contained in this 113K GI, which may diversify the metabolic process of 05HAS68 and ensure its survival in natural habitats.

Aside from the differences in VAFs and GIs, genomic segment shifts may also contribute to the diverse characteristics of *S. suis*. As shown in Figure 2A, the GC skew of the 05HAS68 genome is evidently unbalanced and unsymmetrical. A large-scale genomic rearrangement event occurred around the *ori/ter* axis of 05HAS68, resulting in an “X shape” compared with the genomes of 05ZYH33 and 98HAH12. However, the breakpoints of this genomic rearrangement are located on the transposase genes (positioned from T1 to T8, Fig. 3). Nakagawa *et* *al*. previously demonstrated that unbalanced genome is prone to generate DNA rearrangements in the M3 strain of *S. pyogenes*, which is caused by the loss or acquisition of prophages^29^. A clear “X-shaped” genomic rearrangement exists across the replication axis, and is located between the two *rrn*-*comX* regions and between two prophage coding regions of the M3 strain^29^. In some cases, prophages function as anchoring points for genomic recombination reactions that contribute to the diversification of the bacterial genome architecture^30^. Comparing the genome of 05HAS68 with 05ZYH33 and 98HAH12, we found that 05HAS68 contains 253 unique genes, in which there are four prophage-related gene clusters. We believe that the transposase and/or prophage-related genes may have mediated the genomic rearrangements and created the genomic discrepancies in the 05HAS68 genome.

We also investigated the evolutionary relationships among the *S. suis* strains used in our study. Several *S. suis* housekeeping genes, such as *16S rRNA*, *cpn60*, and *rpoB* have previously been used in conventional phylogenetic approaches^31^,^32^. However, the sequence differences of these genes among *S. suis* strains are too subtle to perform phylogenetic analyses. Thus, we selected the *ITS* gene sequences for intra-species differentiation analyses of *S. suis* according to the methods used in *Mycobacterium abscessus*^33^. The results showed that 05HAS68 is divergent from the virulent strains in evolutionary relationship, which is consistent with the conclusion that the avirulent strain 05HAS68 shows amounts of genomic discrepancies with the highly virulent strains (Fig. 5). Moreover, a previous study suggested that *S. suis* is the lineage with the highest level of evolutionary selection pressure in the genus *Streptococcus*, displays the highest amount of gene gain and gene loss, and presents greatest amount of genomic structure variation^34^. Thus, we believe that the 05HAS68 genome has not reached an ideally balanced genome architecture, and genomic rearrangements will continue to occur in the *S. suis* genome.

In summary, we report the sequencing and analysis of a native avirulent SS2 strain, which is divergent from highly virulent *S. suis* strains in biological and genomic properties. Transposase genes and/or prophage-related genes in 05HAS68 might be responsible for the large-scale genomic rearrangement and the unbalanced genomic structure of this strain, while loss of VAFs and the 89K PAI may contribute to the virulence differentiation from the highly virulent SS2 strains. Although it was protective against highly virulence strains, 05HAS68 may not be directly used as a candidate antibacterial vaccine at present, but we still believe that 05HAS68 has important implications for the development of an effective vaccine to protect humans and animals against *S. suis* infection. Moving forward, we will endeavor to reconcile the immunogenicity and immunization side effects of 05HAS68 inoculation for the vaccine development.

## Methods

### Bacterial strains and culture conditions

A new *S. suis* isolate was collected from the tonsil of a healthy pig housed in an abattoir in the affected areas of Jiangsu Province, China, in 2005. The isolate was designated as 05HAS68. Isolated colonies were grown on Todd-Hewitt broth (Difco Laboratories) supplemented with 1% yeast extract (THY), and incubated at 37°C without agitation^11^. If required, strains were cultured on THY agar plate (1.5% w/v) containing 6% (v/v) sheep blood and incubated at 37°C for 48 h. The bacterial strains used in this study are listed in Table 1.

### Biological properties identification of 05HAS68

Morphological identification of 05HAS68 was performed by Gram staining using an optical microscope^7^, and the bacterial capsule was observed through transmission electron microscopy as described by Charland *et* *al*.^14^. Serological typing of 05HAS68 was performed by agglutination test using specific *S. suis* type 2 antiserum (Statens Serum Institut, Denmark). Biochemical reactions were performed with BioMerieux VITEK 2 Systems Version 05.04. The 16S rRNA classification was performed according to a standard procedure using the following PCR primers: P1 5'-AGA GTT TGA TCC TGG CTC AG-3' and P2 5'-GGT TAC CTT GTT ACG ACT T-3'.

### Titration of hemolytic activity

Hemolysis of SS2 strains were analyzed as described by Pan *et* *al.*^35^, with slight modifications. Briefly, 150 μl serial twofold-diluted culture supernatants of 05HAS68 or 05ZYH33 were prepared with THY medium. Subsequently, 150 μl of 2% fresh pig red blood cells in 1 × phosphate-buffered saline (PBS) was mixed with the diluted bacterial supernatants. The mixed cultures were incubated for 2 h at 37°C and then centrifuged at 600 × g for 10 min. The supernatant (150 μl) was transferred to a microplate and read at 540 nm in a Model 680 Microplate Reader (Bio-Rad).

### Whole blood killing assays

Blood killing assays were similar to a previously published study^36^. Briefly, 300 μl of fresh heparinized pig blood was mixed with 100 μl THY containing 10^4^ CFU of freshly diluted logarithmic phase *S. suis* suspensions. The mixtures were incubated at 37°C for 3 h with gentle agitation, and 100 μl of cultured mixture was inoculated on THY agar plate for enumeration of surviving bacteria.

### Bacterial adherence assays

*S. suis* cultures (05HAS68 and 05ZYH33) were labeled and harvested as previously described^37^. The human laryngeal epithelial cell line Hep-2 (CCTCC GDC004) was utilized to perform bacterial adherence experiments, and cells were cultured to monolayer at 37°C with 5% CO_2_. As described by Hu *et* *al.*^38^, cells were infected with labeled bacteria (ratio 1:100) and then incubated at 37°C for 2 h with gentle agitation. Following incubation, 4% (w/v) paraformaldehyde in PBS was added to each tube to fix cells. The tubes were gently inverted several times and were analyzed using the flow cytometer.

### Virulence studies and protective effect tests

Virulence evaluation and protective effect tests of 05HAS68 were performed as described by Li *et* *al.*^8^, with slight modifications. Briefly, 36 three-week-old SPF piglets (6 piglets/group in random) were injected intravenously through the ear with different bacterial suspensions in a dose of 10^8^ CFU/piglet. For the virulence studies, three groups of piglets were directly injected with 05HAS68, 05ZYH33, or 98HAH12, respectively. The infected piglets were carefully monitored for clinical symptoms more than one month, such as fever (> 41°C), appetite, daily activity, and their survival time. All animal experiments were performed with the approval of the local ethics committee.

To evaluate the protective effect of 05HAS68, two groups of piglets were simultaneously inoculated with 05HAS68, and were given the same dose of bacterial suspensions for booster on day 14. Subsequently, these two groups were challenged with 05ZYH33 or 98HAH12 on 28. To prove the protective effect of 05HAS68, we used an attenuated SS2 strain Δ*salKR* (an engineered avirulent mutant with the SalKR two-component system knockout from 05ZYH33) as a control to repeat the protective experiment as described above.

### DNA extraction

Genomic DNA of 05HAS68 was extracted using a Wizard® genomic DNA purification kit (Promega) according to the manufacturer's instructions. The quality and quantity of extracted DNA were measured using a Qubit® 2.0 fluorometer (Life Technologies). The extracted DNA (≥ 500 ng/µl) was used for whole genome sequencing and PCR verification. All DNA samples were stored in distilled H_2_O at −20°C.

### High-throughput genome sequencing and assembly

Whole genome sequencing was performed by shotgun and mate-pair sequencing methods using an Ion Torrent Personal Genome Machine. The genomic DNA of 05HAS68 was used to construct random shotgun and mate-pair (4 kb) sequencing libraries with genome coverage of 114× and 127×, respectively. Sequences were preliminarily assembled using 454 Newbler v2.8 and CLC genomics workbench. To fill the intra-scaffold gaps, we used mate-pair sequencing information to retrieve the read pairs with one read aligned to the contigs and another read in the gap region. These scaffolds were assembled according to the reference genome of the *S. suis* strain 05ZYH33 (NC_009442), using the Move contigs program in Mauve v 2.3.1^39^. The gaps were closed by primer walking and sequencing of the PCR products.

### Genome annotation

Open reading frame (ORF) prediction was performed using Glimmer version 3.02^40^ and GeneMark^41^ software, and the results were amalgamated. Rapid annotation using subsystem technology was applied for genome annotation^42^. Each coding sequence was annotated by aligning to the non-redundant protein, Cluster of Ortholog Genes, and Kyoto Encyclopedia of Genes and Genomes databases. The ORFs without a reliable hit to any other protein were automatically annotated as “hypothetical proteins.” tRNA and rRNA predictions were conducted with tRNAScan^43^ and RNAmmer^44^, respectively. The genome sequence feature information was visualized using the CGView genome visualization program^45^. The annotated *S. suis* 05HAS68 genome sequence and its annotation information were deposited in GenBank (http://www.ncbi.nlm.nih.gov/genbank/) under accession number CP006930.

### Whole-genome alignment and comparative genomics analyses

The progressive Mauve program in Mauve v2.3.1^39^ and Artemis Comparison Tool^46^ were used for whole-genome alignment among two or more *S. suis* genomes. Further analysis of differential genes in the *S. suis* genomes were accomplished using the BLASTp program, with a maximum expectation value of 1 × 10^−6^ and minimum score of 50. MLST classification was performed based on seven housekeeping genes (*aroA*, *cpn60*, *dpr*, *gki*, *mutS*, *recA*, and *thrA*) through the MLST website (http://www.mlst.net/). The putative virulence genes of 05HAS68 were identified with the BLASTp program using the same statistical limit as above or by comparing with the Virulence Factors Database^47^. GIs of 05HAS68 were determined with IslandViewer^48^. Prophage-related genes and CRISPR were identified using Prophage Finder^49^ and CRISPRFinder^50^, respectively.

### Phylogenetic analysis

To construct a phylogenetic tree, we extracted 16S–23S *ITS* gene sequences from all *S. suis* genomes used in the study. The extracted sequences were aligned using the ClustalW algorithm^51^. The phylogenetic tree was constructed using MEGA5^52^ through the unweighted pair-group method with arithmetic means and 1000 bootstraps. The data of the 11 *S. suis* genomes used for phylogenetic tree construction are listed in Table 1. *S. suis* strain D12 (serotype 9) was used as the out-group of phylogenetic tree construction.

### Ethics statement

All piglet experiments in this study were approved by the Ethics Committee of the Third Military Medical University and performed in accordance with the relevant guidelines and regulations. The welfare of the piglets was adequately protected, and efforts were made to minimize suffering and distress to the piglets.

### Statistical analyses

All of the experiments were biologically repeated more than three times, independently. Where appropriate, the data were analyzed using Student's *t*-test, and a value of *P* < 0.05 was considered significant.

## Author Contributions

F.H., J.T. and X.Y. conceived and designed the experiments; X.Y., M.L., J.W., C.W., D.H., F.Z., X.P. and Y.Z. performed the experiments; X.Y., M.L., Y.T. and L.H. analyzed the data; F.H. and X.Y. wrote the paper.

## Supplementary Material

Supplementary Information Supplementary information

## Figures and Tables

**Table 1:** Characteristics of *Streptococcus suis* genome analyzed in this study

Strain	Serotype	MLST type	Refseq	Size(bp)	GC(%)	Gene	Protein
05HAS68	2	ST28	CP006930	2,176,073	41.2	2077	2009
05ZYH33	2	ST7	NC_009442	2,096,309	41.1	2254	2186
98HAH12	2	ST7	NC_009443	2,095,698	41.1	2253	2185
A7	2	ST7	NC_017622	2,038,409	41.3	2064	1974
BM407*	2	ST1	NC_012926	2,146,229	41.1	2136	1947
GZ1	2	ST1	NC_017617	2,038,034	41.4	2047	1977
P1/7	2	ST1	NC_012925	2,007,491	41.3	2011	1824
S735	2	ST1	NC_018526	1,980,887	41.4	1950	1882
SC070731	2	ST7	NC_020526	2,138,568	41.2	2011	1933
SC84	2	ST7	NC_012924	2,095,898	41.1	2068	1989
D12	9	Not defined	NC_017621	2,183,059	41.3	2190	2078

*having a plasmid.

**Table 2:** Comparison of predicted virulence-associated factors in 05HAS68, 05ZYH33, and 98HAH12

		Strains		
Gene description		05HAS68	05ZYH33	98HAH12
**Adhesin**				
	Agglutinin receptor	+	+	+
	Fibronectin binding protein (FBP)	+	+	+
	Muramidase-released protein (MRP)	−	+	+
	*Streptococcal* hemagglutinin	+	−	−
**Proteinase**				
	Autolysin	+	+	+
	Choline-binding protein D (CbpD)	+	+	+
	Enolase	+	+	+
	Extracellular protein factor (EPF)	−	+	+
	Glutamate dehydrogenase (GDH)	+	+	+
	Histidine triad cell surface-associated protein (HtpS)	+	+	+
	Hyaluronidase (Hyl)	−	+	+
	Sortase A	+	+	+
**Hemolysin**				
	Hemolysin III	+	+	+
	Putative hemolysin	−	+	+
	Suilysin (SLY)	−	+	+
**Others**				
	LTA D-alanylation (DltA)	+	+	+
	PlcR family transcriptional regulator (PlcR)	−	+	+
	Virulence-associated protein E	−	+	+
**TCSTS**				
	CiaRH	+	+	+
	IhK/Irr	+	+	+
	NisK/NisR	−	+	+
	SalK/SalR	−	+	+
	VirR/VirS	−	+	+
	CovR	+	+	+
	RevS	−	+	+
