# Supplementary material for: Isolation and characterization of a native avirulent strain of Streptococcus suis serotype 2: a perspective for vaccine development
Source: Sci Rep. 2015 Apr 20;5:9835. doi: 10.1038/srep09835 (PMC4402706; doi:10.1038/srep09835)
Supplement: Supplementary Information [file srep09835-s1.pdf]

Isolation and characterization of a native avirulent strain of *Streptococcus suis* serotype 2: a perspective for vaccine development

Xinyue Yao[1], Ming Li[1], Jing Wang[2], Changjun Wang[2], Dan Hu[2], Feng Zheng[2], Xiuzhen Pan[2], Yinling Tan[1], Yan Zhao[1], Liwen Hu[1], Jiaqi Tang[3*] & Fuquan Hu[1*]

[1]Department of Microbiology, Third Military Medical University, Chongqing, 400038, China;

[2]Department of Epidemiology, Research Institute for Medicine of Nanjing Command, Nanjing 210002, China;

[3]PLA Research Institute of Clinical Laboratory Medicine, Nanjing general hospital of Nanjing Military Command, Nanjing 210002, China

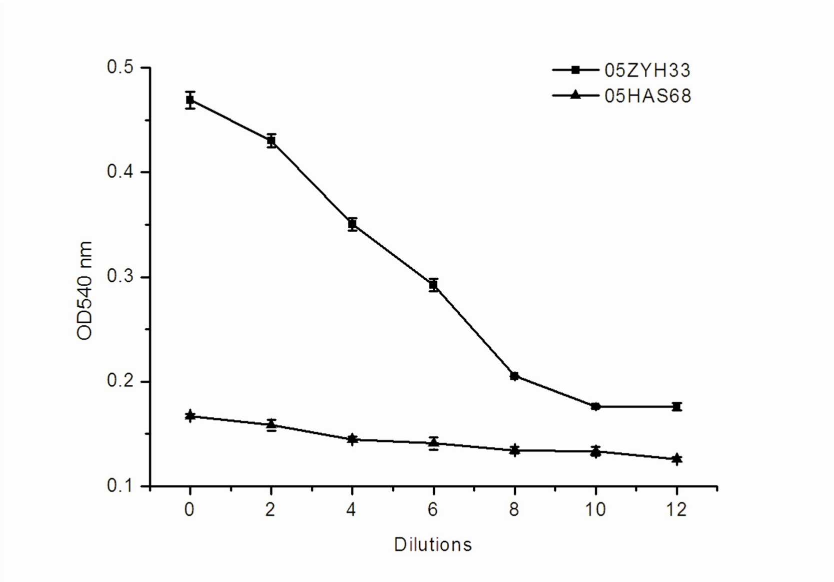

Figure S1 | Titration of hemolytic activities of *S. suis* culture supernatants. The horizontal line indicates the dilution ratio of hemolysin. Optical density was set at 540 nm.

Table S1 | Four Prophage-related gene clusters and a CRISPR structure in *S. suis* 05HAS68 genome

Classification	ID	Start	End	Description
Prophage 1	HAS68_0394	437118	435961	Integrase
	HAS68_0395	438046	437315	hypothetical protein
	HAS68_0396	438477	438094	Phage protein
	HAS68_0397	439032	438484	Phage transcriptional regulator
	HAS68_0398	439049	439249	Cro-like phage transcriptional repressor protein
	HAS68_0399	439319	439687	hypothetical protein
	HAS68_0400	440013	440261	hypothetical protein
	HAS68_0401	440408	440749	hypothetical phage protein
	HAS68_0402	440749	441228	Phage protein
	HAS68_0403	441517	441915	hypothetical protein
	HAS68_0404	441884	443056	hypothetical protein
	HAS68_0405	443130	443396	Phage protein
	HAS68_0406	443406	444104	Phage essential recombination function protein, Erf
	HAS68_0407	444397	444795	Single-stranded DNA-binding protein
	HAS68_0408	444806	445633	putative replication protein
	HAS68_0409	445617	446996	hypothetical protein
	HAS68_0410	447505	447813	Phage protein
	HAS68_0411	447815	448030	hypothetical protein
	HAS68_0412	448219	448533	Phage protein
	HAS68_0413	448606	449040	Phage protein
	HAS68_0414	449691	449936	Phage-associated protein
	HAS68_0415	449933	451198	Phage portal protein; Phage capsid and scaffold
	HAS68_0416	451191	452411	Phage protein
Prophage 2	HAS68_0760	831310	830147	Integrase/recombinase, phage associated
	HAS68_0761	831939	831394	Transcriptional regulator, XRE family
	HAS68_0762	832464	832739	hypothetical protein
	HAS68_0763	832729	832932	hypothetical protein
	HAS68_0764	832925	833128	hypothetical protein

	HAS68_0765	833139	833405	hypothetical protein
	HAS68_0766	833579	833794	hypothetical protein
	HAS68_0767	833772	834602	Chromosome replication initiation protein DnaD
	HAS68_0768	834618	835463	Helicase loader DnaI
	HAS68_0769	835931	836371	hypothetical protein
	HAS68_0770	837081	837467	hypothetical protein
	HAS68_0771	837873	838325	hypothetical protein
	HAS68_0772	838437	838646	hypothetical protein
	HAS68_0773	839753	839974	RelB/StbD replicon stabilization protein (antitoxin to RelE/StbE)
	HAS68_0774	839976	840236	RelE/StbE replicon stabilization toxin
	HAS68_0775	840403	841044	Predicted hydrolase (HAD superfamily)
Prophage 3	HAS68_1309	1435277	1434540	Phage lysin
	HAS68_1310	1435802	1435374	Phage endolysin
	HAS68_1311	1436150	1435806	hypothetical protein
	HAS68_1312	1436368	1436153	conserved domain protein
	HAS68_1313	1441674	1436368	Phage hyaluronidase
	HAS68_1314	1442366	1441671	Phage-associated protein
	HAS68_1315	1444948	1442366	Phage minor tail protein
	HAS68_1316	1445321	1444938	Phage protein
	HAS68_1317	1445596	1445318	conserved hypothetical protein - phage associated
	HAS68_1318	1446194	1445607	Phage major tail protein
	HAS68_1319	1446545	1446210	Phage-associated protein
	HAS68_1320	1446781	1446542	Phage-associated protein
	HAS68_1321	1447112	1446774	Phage-associated protein
	HAS68_1322	1447347	1447099	Phage protein
	HAS68_1323	1448607	1447717	Phage major capsid protein
	HAS68_1324	1449072	1448611	Phage capsid and scaffold
	HAS68_1325	1450571	1449156	Phage terminase
Prophage 4	HAS68_1986	2151776	2151324	hypothetical protein
	HAS68_1987	2152408	2151950	hypothetical protein
	HAS68_1988	2153236	2152421	hypothetical protein
	HAS68_1989	2153507	2153223	hypothetical protein
	HAS68_1990	2153835	2153497	hypothetical protein
	HAS68_1991	2154196	2153993	hypothetical protein
	HAS68_1992	2154411	2154184	hypothetical protein
	HAS68_1993	2155623	2155012	prophage ps3 protein 13
	HAS68_1994	2156397	2155651	kilA protein, putative phage-related DNA binding protein
	HAS68_1995	2156643	2156416	conserved hypothetical protein - phage associated
	HAS68_1996	2156797	2157510	Phage transcriptional repressor
	HAS68_1997	2157874	2158704	hypothetical protein
	HAS68_1998	2158869	2160041	Integrase/recombinase, phage associated
CRISPR	HAS68_0813	881857	885063	CRISPR-associated protein, Csn1 family

HAS68_0814	885035	885979	Mobile element protein
HAS68_0815	886443	887357	CRISPR-associated protein Cas1
HAS68_0816	887369	887677	CRISPR-associated protein Cas2
HAS68_0817	887674	888720	CRISPR-associated protein Cas7
DR 1			GTTTTTGTACTCTCAAGATTTAAGTAACAGTAAAAC
Spacer 1	888817	888845	TGGCCAAACGTGGGATCAAAACCATGTTG
DR 2			GTTTTTGTACTCTCAAGATTTAAGTAACAGTAAAAC
Spacer 2	888882	888911	CTAAGATAAATAATATCCCAATTAATACTG
DR 3			GTTTTTGTACTCTCAAGATTTAAGTAACAGTAAAAC
Spacer 3	888948	888977	TAATTATCTCTTTGGACCGCATCGTGGCCA
DR 4			GTTTTTGTACTCTCAAGATTTAAGTAACAGTAAAAC
Spacer 4	889014	889042	GGATTATTGAGTGGGTTAACATCTGCAGG
DR 5			GTTTTTGTACTCTCAAGATTTAAGTAACAGTAAAAC
Spacer 5	889079	889108	AACATCAATGTATTTCTTTTCGACCTTGTC
DR 6			GTTTTTGTACTCTCAAGATTTAAGTAACAGTAAAAC
Spacer 6	889145	889174	CACTTCCTTACGCTACTAATTTTCAGTTAA
DR 7			GTTTTTGTACTCTCAAGATTTAAGTAACAGTAAAAC
Spacer 7	889211	889240	CTTCTGGTAGTTATCCCCATAACATTTGTA
